# Bibliometric analysis and evidence of clinical efficacy and safety of digital pills

**DOI:** 10.3389/fphar.2023.1023250

**Published:** 2023-01-23

**Authors:** Olena Litvinova, Elisabeth Klager, Andy Wai Kan Yeung, Nikolay T. Tzvetkov, Oliver Kimberger, Maria Kletecka-Pulker, Harald Willschke, Atanas G. Atanasov

**Affiliations:** ^1^ National University of Pharmacy of the Ministry of Health of Ukraine, Kharkiv, Ukraine; ^2^ Ludwig Boltzmann Institute Digital Health and Patient Safety, Medical University of Vienna, Vienna, Austria; ^3^ Division of Oral and Maxillofacial Radiology, Applied Oral Sciences and Community Dental Care, Faculty of Dentistry, The University of Hong Kong, Hong Kong, Hong Kong SAR, China; ^4^ Department of Biochemical Pharmacology and Drug Design, Bulgarian Academy of Sciences, Institute of Molecular Biology “Roumen Tsanev, Sofia, Bulgaria; ^5^ Department of Anaesthesia, Intensive Care Medicine and Pain Medicine, Medical University of Vienna, Vienna, Austria; ^6^ Institute for Ethics and Law in Medicine, University of Vienna, Vienna, Austria; ^7^ Institute of Genetics and Animal Biotechnology of the Polish Academy of Sciences, Jastrzebiec, Poland

**Keywords:** digital pill, ingestible sensor, efficiency, safety, medication adherence, clinical trials

## Abstract

**Objectives:** Digital pills are new technologies that aim to improve healthcare by increasing medication adherence. The aim of the work was a bibliometric analysis of clinical studies of digital pills and an assessment of the level of evidence of their effectiveness, safety, and prospects for the future.

**Materials and Methods:** The studies were conducted using online databases such as ClinicalTrials.gov, Dimensions, and Web of Science for the period January 2012 to July 2022. The VOSviewer tool for building and visualizing bibliometric networks was used.

**Results:** Bibliometric analysis of the scientific literature revealed that over the past 10 years, the number of publications about digital pills has noticeably increased, which indicates the increasing importance of this field of knowledge. The leading positions in this area are occupied by scientists from the United States, the United Kingdom, and India. Sources of financial support for authors of publications in the field of digital pills are funds from leading developer companies, budget allocations, and funds from non-commercial organizations. Public-private partnerships are an important path to develop and implement digital pills. The four main clusters of digital pill studies were highlighted and visualized: efficacy and safety analysis for serious mental disorders; treatment and costs of tuberculosis therapy; features of the treatment of diabetes, cardiovascular diseases, and AIDS; and usage monitoring. Available publications demonstrate the efficacy potential and safety of digital pills. Nevertheless, the effects of digital pills have not yet been fully studied.

**Conclusion:** Priority areas for future research are further randomized controlled clinical trials and meta-analyses, which are necessary for a high level (I level) of evidence for therapeutic applications of digital pills, as well as pharmacoeconomic studies.

## 1 Introduction

One of the areas of promoting health is the use of digital tools for the provision of medical care. The implementation of digital therapeutics and digital care products in health institutions is intended to speed up the exchange of information in the system of quality control of medical care and reduce risks and uncertainties. The medical industry is closely related to the accumulation and processing of significant amounts of information. The effectiveness of first aid and further treatment of the patient depend on the quality of methods of working with information.

Digital pills with ingestible sensors are an effective tool for organizing the provision of quality medical care by providing a doctor with timely and qualified information support and increasing patient adherence to treatment (Baumgartner et al., 2021). Digital pills have built-in sensors that, when used in conjunction with the software of devices like tablets and smartphones, can track the progress of pharmacotherapy. Low patient compliance (medication opt-out) is a significant barrier for all areas of medicine; hence, such monitoring is important.

Examples of well-known digital pills with ingestible sensors are the Ingestible Event Marker sensor (made by Proteus Digital Health, California, USA) and the ID-CapTM system (made by EtectRx, Florida, USA), which received the FDA’s approval (Flore, 2021).

The analysis of data from clinical trials of digital pills is the focus of many scientists and doctors of various specialties (Hafezi et al., 2015; Alipour et al., 2020; Mukherjee et al., 2022). The following types of effects from their implementation into medical practice are distinguished: increasing adherence to treatment of patients; improving the effectiveness and safety of therapy; reducing the cost of treatment and hospital stays; improving the quality of medical care; reducing medical errors; remote access to clinical data; providing mobile clinical monitoring; transparency of patient data, *etc.*

Currently, it can be stated that digital pills are used in various fields of medicine. An analysis of the literature shows that a number of papers focus on experimental clinical studies of them in people with mental health symptoms, monitoring the treatment regimen after transplantation, including pediatric; when taking opioids in people with acute pain; for the treatment of high-risk cardiovascular disease, uncontrolled hypertension, and type 2 diabetes; tuberculosis, hepatitis C, and AIDS (Au-Yeung et al., 2011a; Belknap et al., 2013; Eisenberger et al., 2013; DiCarlo et al., 2016; Flores et al., 2016; Noble et al., 2016; Peters-Strickland et al., 2016; Profit et al., 2016; Chai et al., 2017a; Chai et al., 2017b; Frias et al., 2017; Moorhead et al., 2017; Thompson et al., 2017; Holender et al., 2018; Triplett et al., 2019; Bonacini et al., 2020; Daar et al., 2020; Kamal et al., 2020; Sulkowski et al., 2020).

However, critical analysis of clinical trial outcomes requires attention to the challenges clinicians face, as well as questions that have yet to be answered and final decisions about the benefits and risks of using digital pills. The challenges clinicians face include the accuracy and interpretation of the data they receive, a lack of comparative randomized trials of digital pills with non-digital dosage forms, including pharmacokinetics, a small number of participants in clinical trials, and ethical questions. Following below is short discussion of selected publications discussing these problems in more detail.

Chai *et al.* systematized 18 pilot clinical trials of digital pills in the treatment of HIV, hepatitis C, solid organ transplantation, tuberculosis, schizophrenia, cardiovascular disease, and acute fractures (Chai et al., 2022). Studies typically included up to 50 patients, some between 120 and 288 individuals, and they lasted from 1 week to 6 months. The authors draw the conclusion that digital pills might improve medication adherence and treatment outcomes. However, randomized controlled clinical trials are necessary to compare various treatments quantitatively, which will increase the level of evidence of their effectiveness and safety.

Martani *et al.* analyzed the above 18 clinical trials in terms of ethical aspects (Martani et al., 2020). The authors note the importance of confidentiality in the use of digital pills, as well as the small size of the studied samples, suggesting a low level of evidence.

Other authors also address the ethical challenges of using digital pills. Thus, digital pill therapy is compared by Rosenbaum to “swallowing a spy,” with no discernible therapeutic benefit for patients (Rosenbaum, 2018). The sociologist Hatch called Abilify’s MyCite digital pills a “killer app” aimed at making a profit (Hatch, 2018).

Digital pills should not be seen as a simple change in how medications are taken, state Chevance *et al.* (Chevance et al., 2022). Instead, they should be seen as a complicated intervention that requires rigorous testing before being extensively employed in everyday clinical practice. The authors also stress the significance of an ethical and legal framework in order to secure the legal and moral possession and use of medicine.

Cosgrove *et al.* criticize the efficacy and insufficient comparative studies of digital pills in relation to non-digital dosage forms and placebo, as well as reflecting on their high financial burden (Cosgrove et al., 2019). According to the authors, there were no randomized clinical trials contrasting digital aripiprazole with a non-digital formulation, other comparators, or a placebo. There was no proof that the aripiprazole digital form had higher compliance than the analog version. The authors draw attention to the dishonest strategies used to use evergreen patents to prolong the duration of patent protection and thwart generic competition. Researchers are worried about the approval of a “new” medicine or gadget that may not be as effective or safe but will cost substantially more.

Despite the presence of a significant array of scientific research on digital pills, the problems associated with both analyzing the level of evidence of their effectiveness and safety, identifying leading developers, and assessing the costs and prospects for the future remain poorly understood. The need to solve this range of problems determines both the purpose of the study and its relevance.

At the beginning of the research, it is possible to predict the potential level of development since the dynamics of publication activity are uneven for each section of science, and thus “hot spots” can be identified. They are defined as specific thematic areas in which the amount of work exceeds the industry average. Development in this priority industry increases the chances of it being in demand, but if the work does not contain fundamentally new data, then its commercialization will not be successful. If the research is devoted to a narrow topic that goes beyond the interests of most industry and health researchers, then its commercialization becomes more complicated, regardless of the value of the results obtained. Hence, the aim of the present work was a bibliometric analysis of clinical studies of digital pills and an assessment of the level of evidence of their effectiveness, safety, and prospects for the future.

## 2 Materials and methods

This study involved retrospective, logical, and graphic research methods and content analysis.

**FIGURE 1:**
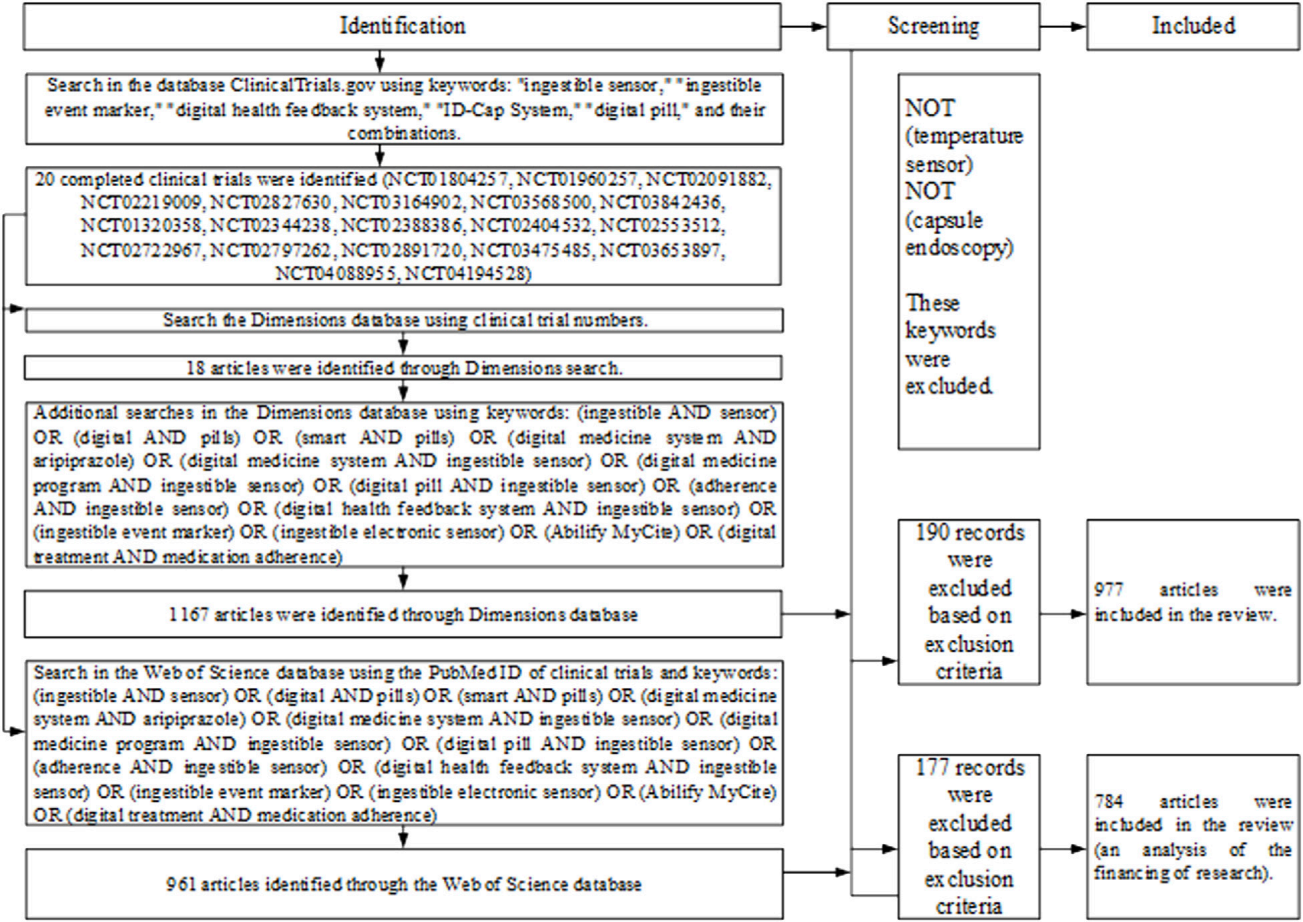
The search strategy.

This study was conducted using online databases, namely ClinicalTrials.gov, Dimensions (https://app.dimensions.ai/discover/publication), and Web of Science. Searches were focused on scientific articles published between January 2012 and July 2022. The search strategy is shown in Figure 1.

In the first stage of research, clinical studies of digital pills were searched in the database ClinicalTrials.gov. The following keywords were used in the search: ingestible sensor, ingestible event marker, digital health feedback system, ID-Cap System, digital pill, and their combinations. By manual screening, relevant clinical trials were identified and their trial numbers were passed to the second stage of the research.

In the second stage of the research, an analysis of publications was carried out using keywords, clinical trial numbers, and their combinations in the Dimensions Database.

In the third stage, original articles and scientific reviews were selected from Dimensions Database documents, which were then considered in more detail based on analytical tools. For bibliometric analysis, the VOSviewer tool for building and visualizing bibliometric networks was used. It can build bibliometric maps based on citation analysis, keyword co-occurrence, co-authorship, and other parameters.

It is possible to deal with a sizable number of publications when using bibliometric data analysis tools, and this makes it possible to spot trends in research activities that are challenging to find without the use of specialized digital tools. Using this strategy, it is possible to pinpoint the direction of research with the necessary level of specificity and depth as well as spot trends in the global publication activity of scientists. Bibliometric analysis is widely regarded as one of the practical techniques that allow one to gain a comprehensive understanding of the issues confronting researchers as well as become acquainted with the potential future directions of scientific inquiry.

In the fourth stage, a detailed analysis of publications related to clinical trials was carried out. The fourth stage evaluated the evidence of efficacy and safety from available publications related to clinical randomized trials and meta-analyses; the identification of gaps in this direction; and problems such as the high cost of the intervention (Cosgrove et al., 2019).

At the fifth stage of research, an analysis of organizations that finance scientists working in the field of digital pills was carried out. The scientometric base of the Web of Science makes it possible to conduct such an analysis. Studies were conducted with keywords as in the Dimensions Database, as well as identifiers of PubMed articles corresponding to clinical trial numbers. The search for organizations financing research in the field of digital pills was carried out using the commercial names of leading developer companies, non-commercial organizations, and state funds.

## 3 Results

### Publications

A search using keywords and clinical trial numbers (as indicated in Figure 1) found 977 results in the Dimensions Database for the field of digital pills.

**FIGURE 2:**
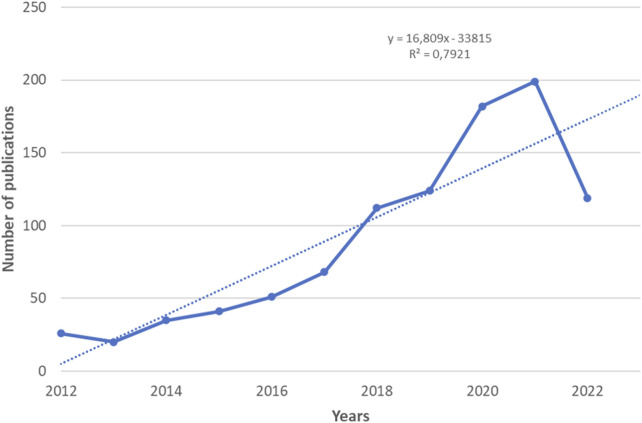
The dynamics of publications related to clinical trials of digital pills and their review papers for the period January 2012 to July 2022.

Figure 2 shows the dynamics of publications related to clinical trials of digital pills and their review papers published between January 2012 and July 2022 in the Dimensions Database. At the same time, the largest numbers of articles were published in 2020 and 2021, covering 18% and 20% of all publications, respectively, for 2012–2022. The number of articles in 2016, 2017, and 2018 compared to 2012 increased by 1.96, 2.61, and 4.3 times, respectively.

Analysis of the data obtained allows us to conclude that the dynamics of publication activity has a positive growth trend during the period under review. The trend model predicts further growth in research in this scientific area. The above confirms intensive research in the area.

### Organizations, countries, scientists, journals, and preprint servers

Harvard University, Proteus Digital Health, Brigham and women’s hospital, University of Manchester, King’s college London are leaders in publishing and scientific activity in the field of digital pills among research organizations (Table 1).

**TABLE 1:** The top leading organizations in publishing and scientific activity in the field of digital pills for the period January 2012 to July 2022.

Organization	Documents	Citations	Total link strength^a^
Harvard university	31	599	359
Proteus digital health	19	566	259
Brigham and women’s hospital	20	472	545
University of Manchester	6	442	17
King’s college London	9	423	46
Rmit university	6	391	22
Monash university	7	290	12
Massachusetts general hospital	20	285	504
University of California, San Francisco	19	273	147
Beth Israel deaconess medical center	18	234	421
Brown university	20	222	298
Stanford university	9	222	68
Otsuka (US)	15	201	116
University of Washington	7	189	78
Miriam hospital	10	56	347

^a^ The Total link strength attribute indicates the total strength of the citation links with organizations.

Researchers from the USA contributed the most to the development of theories about digital pills, publishing 315 papers (315/977, or 32.24% of the total number of publications on the problem under study). Researchers from the United Kingdom came in second with 101 papers (101/977, or 10.33%), and researchers from India came in third with 46 papers (46/977, or 4.71%) (Table 2).

**TABLE 2:** The top leading countries in publishing and scientific activity in the field of digital pills for the period January 2012 to July 2022.

Country	Documents	Citations	Total link strength^a^
United States	315	4,169	108
United Kingdom	101	1,417	75
India	46	313	18
Australia	36	785	17
China	34	248	18
Italy	30	315	13
Canada	25	369	23
Netherlands	22	258	29
Germany	20	338	11
South Africa	20	95	31

^a^ The Total link strength attribute indicates the total strength of the co-authorship links of a given country with other countries.

Leading scientists involved in clinical research of digital pills and the systematization of the obtained results are shown in Table 3. The high citation of these authors is revealed. The most productive authors in this context were Giovanni Kenneth Hugh Mayer (US), Peter Ray Chai (US), Edward W Boyer (US), Rochelle K Rosen (US), Conall Michael O’Cleirigh (US), Yoona A Kim (US), Timothy S Peters-Strickland (US), and Lorenzo Angelo Dicarlo (US).

**TABLE 3:** Leading scientists in publishing and scientific activity in the field of digital pills for the period January 2012 to July 2022.

Name Organization, country	Publications	Citations	Citations mean
Kenneth Hugh Mayer Harvard University, United States	16	180	11.25
Peter Ray Chai Brigham and Women’s Hospital, United States	16	175	10.94
Edward W Boyer Brigham and Women’s Hospital, United States	15	136	9.07
Rochelle K Rosen Brown University, United States	12	88	7.33
Conall Michael O’Cleirigh Massachusetts General Hospital, United States	12	61	5.08
Yoona A Kim Proteus Digital Health, United States	11	202	18.36
Timothy S Peters-Strickland Otsuka (United States), United States	10	230	23.00
Lorenzo Angelo Dicarlo LivaNova (United States) United States	10	311	31.10
Georgia R Goodman Massachusetts General Hospital, United States	10	22	2.20
Achilles Katamba Makerere University, Uganda	8	14	1.75

An analysis of the sources of funding for scientific research in the field of systematization, development, and implementation of digital pills in the Web of Science Database was carried out. For the period 2012–2022, 784 publications were identified. A correlation analysis of the number of publications associated with digital pills by year in the Web of Science and Dimensions Databases showed that there is a high correlation in the indexed studies. The correlation coefficient was found to be 0.95.

An examination of publications in the Web of Science Database pointed that Proteus Digital Health (California, USA), the leading developer of digital pills, is an important player in funding research. In 2% (16) of publications in the field of digital pills, funding by Proteus Digital Health for the period 2012–2022 is indicated as the source. It is noted that Proteus Digital Health, which funded the study, is also the employer of some authors (Belknap et al., 2013).

A digital ingestion tracking system—a sensor known as an “Ingestible Event Marker”—made by Proteus Digital Health was used to create the medicine Abilify MyCite by Otsuka Pharmaceutical Company (Japan). Otsuka Pharmaceutical Company was also involved in financing digital pill developments. Authors of 3.3% (26) of publications noted the receipt of funding by Otsuka Pharmaceutical Company in the field of digital pills.

It should be noted that the company EtectRx (Florida, USA) also funded research in the field of digital pills. EtectRx funded the authors of 1.4% (11) of the publications identified.

Another company funding digital pill research is Gilead Sciences, with 1.8% (14) of publications related to the financing of digital pill development.

It should also be noted the active patenting of technologies related to digital pills by Proteus Digital Health, EtectRx, and Otsuka Pharmaceutical Company, which indicates their commercial interest (Litvinova et al., 2022).

Information on funding by companies for developments in the field of digital tablets is provided for illustration only and not as a limitation. However, the analysis of the data obtained allows us to conclude that financing by development companies is not the main one.

It has been established that the predominant type of financial resources used by researchers in the development of digital pills are proceeds from budget funds, and non-profit organizations. For example, it was revealed that in the field of digital pills, 14% (110) of publications received funding from the National Institutes of Health (NIH, USA), 14.6% (115) from the United States Department of Health and Human Services, 1.4% (11) from the Alliance Healthcare Foundation, and 1.4% (11) from Specialists in Global Health. The budgetary financing of scientific developments is primarily associated with the conduct of fundamental scientific research, which provides technological breakthroughs.

Healthcare and pharmacy are areas where public-private partnerships have become quite widespread (Davis et al., 2021). Thus, the experience of joint financing of the creation of digital pills, both by developer companies and government funds, should be noted (Browne et al., 2018). The development of public-private partnerships to intensify the processes of creating digital pills for the treatment of socially threatening diseases (hepatitis, HIV, oncopathology, tuberculosis, *etc.*) contributes to the pooling of unique innovative resources and the distribution of risks associated with a high degree of uncertainty in the results of innovative activities. Public-private partnerships build the innovative potential of the pharmaceutical industry through the joint activities of organizations, increase the financial resources needed to reform the healthcare system, contribute to the optimization of budgetary expenditures, and increase the efficiency of investments in healthcare, which ultimately contribute to improving the quality of medical and pharmaceutical services.

Hingle M. *et al.* note the benefits of academic and industrial partnerships in digital medicine (Hingle et al., 2019). There are also publications in the field of creating digital tablets, the authors of which are representatives of the university and the developer company (Belknap et al., 2013).

Leading journals and preprint servers presenting publications in the field of digital pills are given in Table 4. They are connected with scientific directions such as Medical and Health Sciences; Public Health and Health Services; Clinical Sciences; *etc.*

**TABLE 4:** Leading journals and preprint servers presenting publications in the field of digital pills for the period January 2012 to July 2022.

Journals and preprint servers	Publications	Citations	Citations Mean
JMIR Preprints	53	3	0.06
Research Square	17	0	-
Journal of Medical Internet Research	15	297	19.80
PLOS ONE	13	285	21.92
JMIR mHealth and uHealth	13	286	22.00
JMIR Research Protocols	9	36	4.00
JMIR Formative Research	9	17	1.89
Journal of Clinical Oncology	9	1	0.11
BMJ Open	8	50	6.25
Trials	7	89	12.71

### The terminological map of publications

We used the VOSviewer program’s method of keyword clustering (terms from “Title and Abstract”). The diameter of the circle indicates how frequently the term is used. The separation between the circles acts as a barometer for the degree of association between terms, which is interpreted as the frequency of terms occurring together frequently. Relationships get stronger the closer they get. Thus, keywords form specific thematic clusters.

**FIGURE 3:**
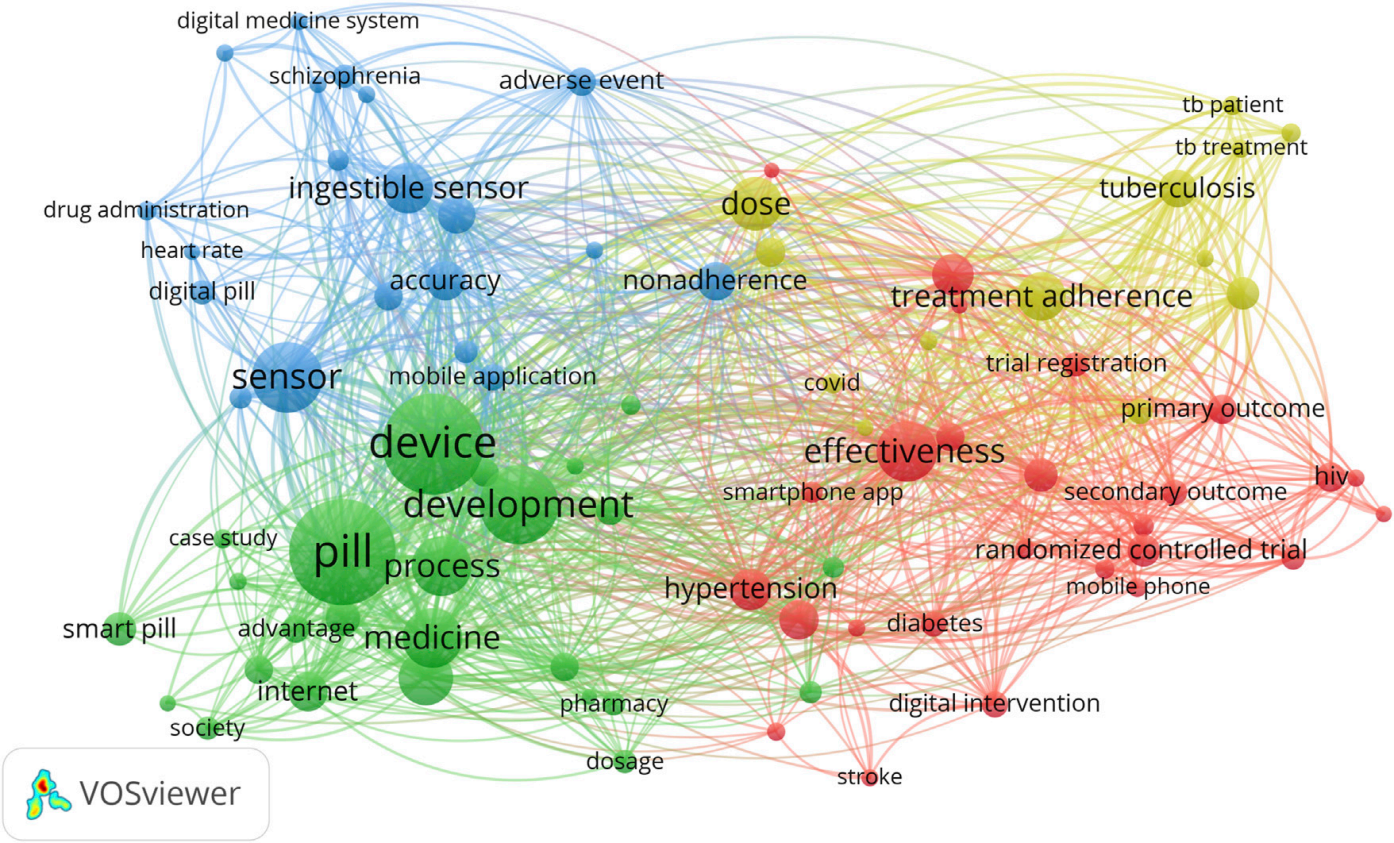
The terminological map of publications in the field of development and research of digital pills for the period January 2012 to July 2022.

On the terminological map in Figure 3, clusters are indicated with various colors; each keyword’s size is determined by the “total link strength” indicator, which measures how strongly a specific keyword is associated with all other phrases; and the lines demonstrate associations between two different keywords.

The degree to which one phrase is used frequently in conjunction with another was evaluated. A specific thesaurus (446 terms) was created to integrate related terms and get rid of keyword typos. Keywords that appear at least 15 times in the sample were chosen in order to construct a terminological map. There are 84 terms in the final set of keywords.

Based on the analysis’s findings, 4 clusters were identified to be related to digital pills and comprised of the subjects of the most referenced authors’ scientific studies.

The first cluster (marked in blue) is devoted to the analysis of the effectiveness and safety of digital pills in patients with severe mental disorders, such as schizophrenia. “Accuracy”, “adverse events”, “aripiprazole”, “caregiver”, “digital health”, “digital medicine system”, “nonadherence”, “safety”, “schizophrenia”, and “serious mental illness” are the main terms of this cluster.

The second cluster (marked in yellow) is related to the treatment and cost-effectiveness issues of using digital pills in patients with tuberculosis. This cluster is represented by the following terms: “cost effectiveness”, “digital adherence technology”, “dose”, “tuberculosis patient”, “tuberculosis treatment”, “treatment adherence”, *etc.*

The third cluster (in red) relates to the features of the treatment of diabetes, cardiovascular diseases, and antiretroviral therapy using digital pills. The most common terms are “antiretroviral therapy”, “HIV”, “cardiovascular diseases”, “blood pressure”, “effectiveness”, “feedback”, “clinical outcome”, “digital intervention”, “hypertension”, “stroke”, “mobile health”, *etc.*

The fourth cluster (marked in green) is connected to the usage monitoring of digital pills. This cluster includes such terms as “advantage”, “adverse effect”, “artificial intelligence”, “elderly person”, “dosage”, “healthcare”, “health outcome”, “innovation”, “society”, *etc.*

These clusters show the links between scientific topics and potential key areas of further research.

The resulting network has a high density, which is expressed by the multiplicity of links between the terms and indicates their high compatibility and occurrence in various studies. The terms given in Table 5 have the greatest weight (according to the occurrence numbers in the analysed literature).

**TABLE 5:** The top keywords in publishing and scientific activity in the field of digital pills for the period January 2012 to July 2022.

Term	Occurrences
Sensor	131
Effectiveness	106
Treatment adherence	76
Hypertension	61
Internet	57
Blood pressure	54
Tuberculosis	53
Smart pill	45
HIV	38
Innovation	35
Adverse event	35
Digital intervention	32
Cost effectiveness	30
Diabetes	30
Schizophrenia	26

The complex nature of the topic of digital pills allows us to conclude that promising areas of research may be at the intersection of two or more clusters.

### The top five most cited publications

Of greatest interest in terms of identifying the most significant and discussed problems in the area of digital pills are the most cited research publications. The works that caused most resonance in academic science are given in Table 6.

**TABLE 6:** The Top five most cited publications for the period January 2012 to July 2022.

Title of article	Authors	Article type, conclusion	Year of publication	Number of citations	Research Area (Journal)
Nonadherence with antipsychotic medication in schizophrenia: challenges and management strategies	Haddad, Peter, Cecilia Brain, and Jan Scott	The review includes the systematization of data on the use of digital pills in patients with schizophrenia, hypertension, heart failure, and tuberculosis The acceptability of “smart pills”, particularly for seriously ill patients, is still unknown and needs more research. These electronic alternatives might eventually become less expensive and more readily accessible in everyday practice as opposed to being restricted to specialized or research contexts. However, it’s crucial to always keep in mind that none of these interventions can replace a thoroughly thought-out treatment plan that has been mutually agreed upon by the patient and the healthcare provider	2014	329	Patient Related Outcome Measures
Metamaterial Inspired Microwave Sensors	Schueler, Martin, Christian Mandel, Margarita Puentes, and Rolf Jakoby	The review includes details on mass flow sensors, differential sensors, wireless strain sensors, and level sensors The upcoming decades will be shaped by sensor systems. They will create sensor networks that can recognize, localize, and keep an eye on parameters related to biological and medical issues	2012	189	IEEE Microwave Magazine
A Human Pilot Trial of Ingestible Electronic Capsules Capable of Sensing Different Gases in the Gut	Kalantar-Zadeh, Kourosh, Kyle J. Berean, Nam Ha, Adam F. Chrimes, Kai Xu, Danilla Grando, Jian Zhen Ou, et al	A pilot trial of an electronic capsule capable of sensing oxygen, hydrogen, and carbon dioxide was conducted The gas-sensing capsule provides a precise and secure method for tracking an individual’s dietary impacts. It has the potential to be utilized as a diagnostic tool	2018	168	Nature Electronics
Effect of Reminder Devices on Medication Adherence: The REMIND Randomized Clinical Trial	Choudhry, Niteesh K., Alexis A. Krumme, Patrick M. Ercole, Charmaine Girdish, Angela Y. Tong, Nazleen F. Khan, Troyen A. Brennan, Olga S. Matlin, William H. Shrank, and Jessica M. Franklin	A randomised clinical trial of medication adherence reminder devices (a pill bottle strip with toggles, a digital timer cap, or a standard pillbox) was carried out The use of low-cost reminder devices by non-adherent patients who were taking up to three drugs to address common chronic illnesses did not increase adherence. If the devices had been combined with interventions to ensure regular usage or had been aimed at people who were even more at risk of nonadherence, they might have been more beneficial	2017	144	JAMA Internal Medicine
Adherence Interventions and Outcomes of Tuberculosis Treatment: A Systematic Review and Meta-Analysis of Trials and Observational Studies	Alipanah, Narges, Leah Jarlsberg, Cecily Miller, Nguyen Nhat Linh, Dennis Falzon, Ernesto Jaramillo, and Payam Nahid	A meta-analysis of 129 studies to ensure adherence to tuberculosis treatment was conducted Adherence strategies include patient education and counseling, rewards and enablers, psychological therapies, reminders and tracers, and digital health technology. Their use improves the outcomes of tuberculosis treatment	2018	131	PLOS Medicine

A study by Haddad *et al.* (Haddad et al., 2014), aimed at investigating the problems of nonadherence with treatment regimens in schizophrenia and management strategies, takes first place in the ranking. According to VOSviewer, this study was cited the most (329 times) from 2014 to the present. The authors note that nonadherence with the treatment regimen is observed in all chronic diseases but is especially dangerous in schizophrenia. Assessing plasma neuroleptic levels for adherence monitoring is challenging. The individual variability of the drug in plasma is high. In addition, metabolism, smoking, and drug interactions affect the concentrations of antipsychotics. The benefits of an ingestible event marker developed by Proteus are noted. The need for further research on electronic monitoring of the use of neuroleptics is emphasized.

The next most cited study is by Schueler *et al.* (Schueler et al., 2012), dedicated to the emergence of “smart sensors” with computing capabilities connected to each other wirelessly. Sensors form networks and identify and monitor various processes, health conditions, *etc.*

The third most cited work by Kalantar-Zade *et al.* (Kalantar-Zadeh et al., 2018) was aimed at directly analysing pilot trials of electronic capsules, providing information on the chemical composition of the intestine. The authors proposed an accurate and safe tool to monitor the effects of diet on people’s digestion, which can be used as a diagnostic tool for the gut. Subject gas profiles were obtained by varying dietary fiber intake and modulating the enzymatic activity of gut microbes. Ultrasound imaging confirmed that the oxygen equivalent concentration profile could be used as an accurate marker of the capsule location.

A significant contribution to the systematization of digital pill representations is made by the study by Alipanah *et al.* (Alipanah et al., 2018). Unlike previously published papers, there has been a systematic review and meta-analysis of 129 studies to ensure adherence to tuberculosis treatment, including directly observed therapy (DOT), training and counselling, financial reward, reminders, psychological support, digital tools, and combinations thereof. Tuberculosis outcomes were found to improve with the use of combined adherence measures. The addition of other compliance interventions to the DOT correlates with reduced mortality rates and higher rates of treatment and recovery. The conducted meta-analysis has limitations. First, contemporary, more integrated approaches to HIV/TB care were not assessed in this review. Second, the studies under review were heterogeneous in their methodology.

A study by Choudhry *et al.* (Choudhry et al., 2017) examines the comparative effects of diverse reminder devices on treatment adherence in 53,480 patients. It was found that reminder devices did not improve patient adherence to treatment. The authors attribute the obtained results to patients’ initial suboptimal adherence and device use difficulties. Further research is needed to implement effective tools to increase adherence to treatment.

The obtained Dimensions Database results were analysed and systematized regarding comparative clinical trials, pharmacokinetic parameters, physicians’ opinions, and the socio-economic aspect of digital tablets with a swallowed sensor. Discussion related to the respective findings is given below.

## 4 Discussion

The data presented indicate that companies and government funding agencies are interested in conducting research on the possibility of using digital pills.

The conducted investigation reveals uses of digital pills in the following fields: cancer, tuberculosis, transplantology, HIV/AIDS, cardiovascular disorders, diabetes mellitus, gastroenterology (including hepatitis C), and pain relief. According to the studies mentioned above, the use of digital pills would boost patient safety, enhance treatment outcomes, promote compliance, shorten hospital stays, and provide mobile clinical monitoring.

It should be noted that the incorporation of information technologies into medicine is undeniably important because missed diagnoses, delayed treatment, and poor adherence to treatment result in disease progression, disability, and, in some cases, death.

The available publications also confirm the safety of Proteus digital pills. The ingestible sensor is made of copper chloride, magnesium, and silicon and signals the patch when it collides with gastric acid. The amount of minerals released into the body is much less than the levels commonly found in a typical diet (Flore, 2021).

Plowman R. S., *et al.*, note that under Section 21 of the Code of Federal Regulations (CFR), Proteus digital pills have been deemed safe and effective for general use by the public in multiple domains, including: performance testing, human factors, electrical safety, electromagnetic compatibility, toxicology, clinical testing, and biocompatibility testing, which meet the special controls of the FDA (Plowman et al., 2018).

However, there is evidence of the side effects of the Proteus digital pills. Peter Chai notes that of the 16 studies that used the Proteus digital pills, 10 studies reported local skin irritation, described as inflammation, at the patch site in 13.2% of participants (Chai et al., 2022).

Studies in the Dimensions Database have revealed that recently, data from pharmacokinetic parameter studies have been obtained for digital pills. This can serve as evidence of bioequivalence relative to non-digital dosage forms or reveal possible benefits.

Thus, comparative pharmacokinetic studies of the traditional form of Truvada (enofovir disoproxil fumarate/emtricitabine) and its encapsulated form (*n* = 24) were carried out in a digital system with an oral sensor (Proteus Digital Health) (Ibrahim et al., 2018). The authors conclude that the investigational formulations are bioequivalent.

Another study (*n* = 49) examined the pharmacokinetics of combined antiretroviral (ARV) medicines encapsulated in an ingestible sensor system (Proteus Digital Health Feedback) (Liu et al., 2020). The following combinations were used as ARVs: emtricitabine (FTC)/tenofovir disoproxil fumarate (TDF); FTC/tenofovir alafenamide (TAF); efavirenz (EFV)/FTC/TDF; abacavir (ABC)/lamivudine (3 TC); dolutegravir (DTG)/ABC/3TC; rilpivirine (RPV)/TAF/FTC; elvitegravir (EVG)/cobicistat (COBI)/FTC/TAF; and bictegravir (BIC)/FTC/TAF. The obtained experimental results were compared with the literature data. The Cmax and AUC values were not statistically significantly different from the literature data for all formulations except for the Cmax of FTC/TDF, the Cmax of BIC, and the Cmax of RPV. The authors attribute the observed deviation in FTC/TDF (Truvada) to participants’ characteristics and fasting/feeding conditions. There is no doubt that further pharmacokinetic studies of digital pills with encapsulated ARVs and traditional forms of ARVs are necessary in order to identify bioequivalence and possible benefits.

The presented data indicates that in pharmacokinetic parameters, encapsulated ARVs with digital pills are not inferior to traditional forms. That is, they are bioequivalent but have advantages in adherence to treatment.

While the increase in adherence when using digital pills was not in doubt, the comparative effectiveness of digital pills and traditional forms has not been sufficiently studied, and it is also important to identify side effects.

In this regard, it is of interest to acknowledge the work of Browne *et al.* on the comparative study of therapy with wirelessly observed therapy using digital pills (WOT) and directly observed therapy (DOT) during the treatment of tuberculosis (*n* = 77) with a combination of isoniazid 150 mg/rifampin 300 mg. 100% of the participants chose to use WOT (Browne et al., 2019). WOT-related adverse events were less than 10% and consisted of minor patches of skin rash and itching. The authors conclude that in terms of accuracy parameters, WOT was equivalent to DOT, and the vast majority of participants preferred digital therapy. This study has a number of limitations, including collecting data only during the continuation phase of tuberculosis therapy. Data are needed on the efficacy of WOT during the intensive phase of tuberculosis treatment. The authors note that DOT is resource-intensive, especially over geographical distances, time-consuming, and represents the single largest cost of tuberculosis treatment.

The use of digital pills for the treatment of tuberculosis is being investigated by other scientists. A quantitative survey was conducted among stakeholders directly involved in tuberculosis management, revealing that digital pills are seen as an acceptable, practicable, and beneficial technology for tracking antitubercular medication adherence (Vaz et al., 2022).

Of special note is the 8-week multicenter study of the efficacy and safety of antipsychotics (aripiprazole, quetiapine, olanzapine, or risperidone) encapsulated in the Proteus digital medical system in patients with schizophrenia, schizoaffective disorder, or the first episode of psychosis (*n* = 55), which had sufficient efficacy and tolerability (Fowler et al., 2021). There is no data on the difference in pharmacological effects of the antipsychotics studied. The study’s potential weakness was its small sample size, particularly in the schizoaffective disorder group (*n* = 10). It might have contributed to the group’s high degree of variability in the percentage of time with satisfactory patch coverage and adherence score. The authors concluded that it required further clinical trials.

Of particular interest also are works that are related to the study of the opinions of doctors regarding digital pills in medical practice.

A survey of 56 tuberculosis control programs in the United States revealed the promise of using directly monitored electronic therapies, including digital pills (Macaraig et al., 2018).

Ruetsch *et al.*, performed a survey of physicians (*n* = 131) prescribing digital pills (Ruetsch et al., 2021). It has been established that doctors are ready to use digital technologies to improve the accuracy of the assessment of compliance with the treatment regimen.

In a pilot study (*n* = 49), an integrated call center is reported to have contributed to the implementation of a digital medical system with aripiprazole in patients with bipolar I disorder, major depressive disorder, and schizophrenia (Kopelowicz et al., 2017).

The issue of using digital pills has not only a medical but also a socio-economic aspect. On the one hand, timely and effective therapy with digital pills combined with a high degree of adherence to treatment significantly reduces the number of patients with disability and severe degrees of disability. Along with this, it should be noted that treatment with digital pills has a high cost. This jeopardizes the possibility of prolonged access for patients to therapy.

In this regard, the study of Forma *et al.* (Forma et al., 2022) involved an online survey on willingness to pay for adherence monitoring tools among individuals (*n* = 184) caring for patients with serious mental disorders. A high preference for the use of digital pills, which monitor not only the use of medicines but also the physical activity of patients, the quality of rest, and mood, has been identified. In addition, respondents reported a willingness to pay $255 more for digital pills *versus* a pill organizer.

According to the literature, there is currently insufficient information on pharmacoeconomic studies of digital pills. The emergence of such information will expand the data on the benefits of digital medicine.

Our analysis revealed that some of the funding for digital pills research is provided by private companies. Since such companies are a stakeholders with financial interests linked to research outcomes, proper disclosure of competing interests in the resulting academic works is of a great importance. From the perspective of the World Health Organisation Commission on Intellectual Property Rights, Innovation, and Public Health, public-private partnerships in general are an effective way to benefit from public and private sector opportunities to address health problems that, alone, neither party can properly address (Commission on Intellectual Property Rights and Innovation and Public Health, 2006). A bright example of a public-private research and development partnership in the European Union is the implementation of projects under the Innovative Health Initiative. The goals of this initiative are to translate health research and innovation into tangible benefits for patients and society and ensure that Europe remains at the cutting edge of interdisciplinary, sustainable, patient-centric health research. In the area of accelerating the development and admission of medicines to the market in the United States, a public-private partnership is being implemented that combines the National Institutes of Health, the US Food and Drug Administration (FDA), pharmaceutical companies, and non-profit organizations. As world experience shows, a clear organizational structure of management and appropriate regulatory support are important conditions for successful scientific research. It should be noted that research transparency and protection against unfair competition are required. These practices are meant to ensure impartiality and prevent conflicts of interest. Consequently, it should be noted the key importance of adhering to established intellectual property policy, ensuring the protection of authors’ and copyright holders’ rights, and using intellectual property while taking into account the interests of all interested parties in conjunction with public interests.

Of particular note is the comparative cost study for direct confirmation of tuberculosis treatment with wirelessly observed therapy using digital pills (WOT) and directly observed therapy (DOT) (Au-Yeung et al., 2011b). Utilizing information from public treatment sources, staff expenses, patient costs, and interview responses, the costs for the two methods of follow-up were compared. In terms of the cost of treatment in a public health facility, the cost of WOT was projected to be 36 percent of the 7-day DOT and 71 percent of the 3-day DOT. WOT was estimated to cost 4% of the 7-day DOT and 8% of the 3-day DOT to treat a patient. The authors come to the conclusion that using WOT to treat M. tuberculosis looks to be much less expensive than using DOT.

A number of clinical trials of digital pills are currently underway. A number of clinical trials are presented in Table 7. It should be noted that most clinical studies are single-arm cohort studies (uncontrolled). It is interesting to observe that none of the studies in Table 7 deals with mental.

**TABLE 7:** Examples of on-going clinical trials of digital pills.

Study number	Study Title	Study Design	Condition or disease	Estimated Enrollment
NCT02800655 (Digital Health Feedback System for Longitudinal Measurement of Medication Adherence During Anti-Retroviral (ARV)Therapy, 2022)	Digital Health Feedback System for Longitudinal Measurement of Medication Adherence During Anti-Retroviral (ARV)Therapy	Prospective single arm open label intervention study	HIV	100
NCT03693040 (Digital Health Feedback System for Longitudinal Monitoring of ARVs Used in HIV Pre-exposure Prophylaxis, 2022)	Digital Health Feedback System (DHFS) for Longitudinal Monitoring of ARVs Used in HIV Pre-exposure Prophylaxis	Prospective single arm open label intervention study	HIV Prevention	100
NCT04065347 (Quantification of Tenofovir Alafenamide Adherence (QUANTI-TAF), 2022)	Quantification of Tenofovir Alafenamide Adherence	Prospective	HIV/AIDS	212
NCT04418037 (DHFS for Medication Adherence Support During Hospital Admissions for Person Living With HIV, 2022)	DHFS for Medication Adherence Support During Hospital Admissions for Person Living With HIV	Prospective single arm open label intervention study	HIV/AIDS	30
NCT05183529 (Monitoring Vital Signs With a Wireless Ingestible Device in Subjects Undergoing Polysomnography, 2022)	Monitoring Vital Signs With a Wireless Ingestible Device in Subjects Undergoing Polysomnography	Single Group Assignment	Sleep	20
NCT05259501 (Remote Methadone Ingestion Surveillance Trial (RMIST), 2022)	Remote Methadone Ingestion Surveillance Trial	Parallel Assignment Arm: tablet-based approach using the ingestible sensor and smart pill bottle Arm: liquid vial-based approach using guided video recording with tamper-aware packaging	Opioid Use Disorder	40

Thus, the possibilities of using digital pills seem very promising for their further testing within the framework of various pathologies.

Nevertheless, the effects of digital pills have not yet been fully studied. Additional randomized controlled clinical trials and meta-analyses are necessary for a high level of evidence for therapeutic investigations (I level) (Burns et al., 2011).

There are a number of restrictions on both the pharmacological and clinical plans connected with their practical application.

These restrictions are defined as follows.

a) scarcity of large-scale comparative randomized clinical trials comparing the efficacy and safety of digital and non-digital forms; data from meta-analyses;
b) there is an insufficient number of pharmacokinetic studies of digital pills confirming their bioequivalence with non-digital dosage forms or identifying benefits;
c) issues related to the high cost of pharmacotherapy;
d) privacy, informed consent, data handling, and ethics issues.

The emergence of such information will expand the therapeutic arsenal of digital medicine.

The growing cost, complexity, and riskiness of innovative projects necessitate the search for new forms of interaction and cooperation between participants in the scientific and private business sphere. The pharmaceutical industry is special, given the depth of its dependence on the knowledge, intensity, and effectiveness of basic research (The 10 biggest science stories of 2022, 2022). In recent decades, the global costs of pharmaceutical companies for innovative medicine development have increased significantly (Ten years on Measuring the return from pharmaceutical innovation 2019, 2022). The listed challenges pose new tasks and problems regarding innovation policy, forcing the most powerful participants in the pharmaceutical industry and the state to look for new mechanisms to support innovative activities.

According to studies, mechanisms of public-private partnership are implemented in the field of digital medicine creation with the participation of many interested parties (states, companies, universities, non-profit organizations). The possibility of combining resources and sharing the financial burden and risks makes such projects more attractive and viable.

In conclusion, it should be noted that the importance of the problem of adherence to treatment will steadily increase in the coming years, which is associated both with the further actualization of cardiovascular disease, diabetes, HIV/AIDS, and tuberculosis in the world and with an increase in life expectancy. Further study, clinical testing, and the implementation into the practice of new effective and safe digital pills for pharmacotherapy will continue to be high priority areas of medicine.

## 5 Conclusion

Bibliometric analysis of the scientific literature revealed that over the past 10 years, the number of publications about digital pills has noticeably increased, which indicates the increasing importance of this field of knowledge. The leading positions in this area are occupied by scientists from the USA, the United Kingdom, and India, and the leaders in publishing and scientific activities among research organizations are Harvard University, Proteus digital health, Brigham and women’s hospital, University of Manchester, King’s college London. Sources of financial resources for authors of publications in the field of digital pills are funds from leading developer companies, budget allocations, and funds from non-commercial organizations. Public-private partnerships are an effective way to develop and implement digital pills. Clearly there is a high need for research on cheaper, non-invasive alternatives to enhance medication adherence, whereby non-profit institutions including public universities should play a more active role in such endeavors.

Using the VOSviewer program, terminology maps were built according to the selected concepts of the respective scientific literature, and their main intersection points were found. The four main clusters of digital pill studies were highlighted and visualized: efficacy and safety analysis for serious mental disorders; treatment and costs of tuberculosis therapy; features of the treatment of diabetes, cardiovascular diseases, and AIDS; and usage monitoring.

An analysis of the five most cited publications on the problems of digital tablets from 2012 to 2022 showed that the most resonant and debatable topics are: strategies for increasing adherence to treatment; types of sensors for digital therapy; and results of clinical studies and meta-analyzes.

Promising directions for further clinical studies of digital pills are: the conduct of randomized controlled trials and meta-analyses to increase the level of evidence of their effectiveness and safety; studying their pharmacokinetic parameters to confirm bioequivalence or to identify the benefit; and pharmacoeconomic analysis.

## Limitations

This study had several limitations. First, we conducted a review with stringent inclusion criteria; this review may have missed non-indexed articles that consider digital pills. Second, because we only included English-language papers, it is possible that we overlooked advancements in ingestible sensor technologies for digital pills that were written about in other languages.
